# Protocol for Community-Based Exercise Training after Discharge from Hospital-Based Stroke Rehabilitation: A Multicenter, Randomized, Parallel-Group, Double-Blind Controlled Pilot and Feasibility Trial

**DOI:** 10.3390/healthcare11162275

**Published:** 2023-08-12

**Authors:** Dongheon Kang, Jiyoung Park, Seon-Deok Eun

**Affiliations:** Department of Healthcare and Public Health Research, National Rehabilitation Center, Ministry of Health and Welfare, Seoul 01022, Republic of Korea; jakekang@korea.kr

**Keywords:** stroke, exercise, rehabilitation, muscle strength, cardiorespiratory fitness

## Abstract

Exercise training participation of patients with stroke in the community after discharge from the hospital has many benefits for physical, social, and psychological rehabilitation and improves their quality of life. However, in the Republic of Korea, studies on stroke survivors who can participate in an exercise training program have not been conducted. This trial aims to investigate the effectiveness of exercise training programs after patients with stroke are discharged from the hospital with a doctor’s note and referred to a community exercise center, as there is a lack of studies on this population. This multicenter, randomized, parallel-group, double-blind controlled pilot and feasibility trial will randomly assign 120 patients with stroke to either 8 weeks of a community-based exercise training program (experimental group) or activities of daily living (control group). The primary outcomes will be muscle strength, cardiorespiratory fitness, body composition, physical performance, and gait. The secondary outcomes will be quality of life and activities of daily living. This study’s results may add new insights into the effectiveness of community-based exercise training programs after patients with stroke are discharged from the hospital with a doctor’s note and referred to a community exercise center. The success of the new exercise training approach could offer valuable information for developing more inclusive protocols for patients with stroke in the future if it proves to be efficacious.

## 1. Introduction

The incidence of stroke has increased over the past few decades, making it a leading cause of death and long-term disability worldwide [1,2]. Stroke is the second and third highest causes of mortality worldwide and in the Republic of Korea, respectively, after cancer and cardiovascular disease [3,4]. In addition, approximately 105,000 people in South Korea experience new or recurrent strokes each year [5]. Progress in early intervention and interventional therapies for stroke has decreased mortality rates and improved survival, making post-discharge care a crucial concern for patients with stroke [3,6,7,8].

Stroke causes muscle atrophy and weakness leading to mobility and functional limitations. Additionally, muscle weakness is related to decreased walking speed and endurance. Decreased cardiorespiratory fitness may also occur, which can negatively impact one’s independence and quality of life [9,10,11,12]. Several epidemiology studies have proven a negative correlation between all-cause mortality and muscle strength and lean mass in both healthy and chronic disease populations [13,14,15,16,17,18]. Having low levels of cardiorespiratory fitness is a significant predictor of all-cause mortality and cardiovascular disease risk, regardless of age and other risk factors [19,20,21,22,23]. It has also been linked to an increased risk of stroke [24,25]. Cardiorespiratory fitness is a crucial factor for predicting the risk of stroke [26].

Given the positive effects of exercise training on these sequelae after hospital discharge, it is necessary to implement exercise training that can significantly improve stroke recovery and rehabilitation [27]. Exercise training involves the intentional participation in planned, structured, and repeated physical activities to improve or maintain physical fitness [28]. Encouraging patients with stroke to participate in physical activity is crucial because low levels of physical activity increase the risk of cardiovascular disease [29]. To ensure successful engagement in physical activity and achieve better cardiorespiratory fitness, functional capacity, and gait, it is crucial to have well-designed exercise programs for patients with stroke, as aforementioned [26]. Numerous meta-analyses have shown that exercise intensity, frequency, type, duration, and volume play vital roles in stroke rehabilitation. These studies have concluded that exercise training can significantly improve rehabilitation outcomes [30,31,32,33,34,35,36].

Patients with stroke require rehabilitation to overcome functional impairments. There is a growing interest in exercise-based rehabilitation, as it improves physical fitness [26,37]. Aerobic and resistance exercises can potentially mitigate stroke-related disabilities through various mechanisms. Exercises that combine aerobic and resistance training are ideal for improving all four components, including muscle strength, cardiorespiratory fitness, functional capacity, and walking ability, compared to any single exercise modality. While aerobic exercise training is effective for improving cardiorespiratory fitness and gait function, resistance exercise training can enhance its effects by increasing muscle strength and physical performance [38,39,40,41]. According to a scientific statement by the American Heart Association, stroke survivors should engage in aerobic and resistance training [42]. However, most people with stroke living in the community after discharge from the hospital are physically inactive or participate in activities that provide little benefit to their recovery [43,44,45]. Participation in exercise is also limited for patients with stroke in community rehabilitation settings because of safety concerns, and new methods are needed to make exercise safer [30,46]. One of these new methods could be to refer patients with stroke to community exercise centers for exercise training with a doctor’s note.

Accordingly, after discharge from the hospital, patients with stroke need to be examined for the efficacy of exercise training programs that consider their physical fitness and health status, including muscle strength, cardiorespiratory fitness, physical performance, and gait, based on their current medical history and doctor’ note. However, in the Republic of Korea, there are no studies on stroke survivors who have received a doctor’s note to participate in an exercise training program linked to an exercise center in the community to safely participate in exercise training after hospital discharge. Therefore, we established a research collaboration, named the Community-Based Rehabilitation Health Promotion Physical Fitness (ReHAPPY), which consists of professionals in rehabilitation and exercise specialists, to determine the effectiveness of exercise training programs after patients with stroke are discharged from the hospital with a doctor’s notes and referred to a community exercise center.

## 2. Materials and Methods

### 2.1. Study Design and Setting

This study is designed as a multicenter, randomized, parallel-group, double-blind controlled pilot and feasibility trial in patients after a first-ever stroke. Trained instructors from the participating centers will conduct the trials. The participants will be randomly assigned to either an intervention program or activities of daily living (ADLs).

**Figure 1:**
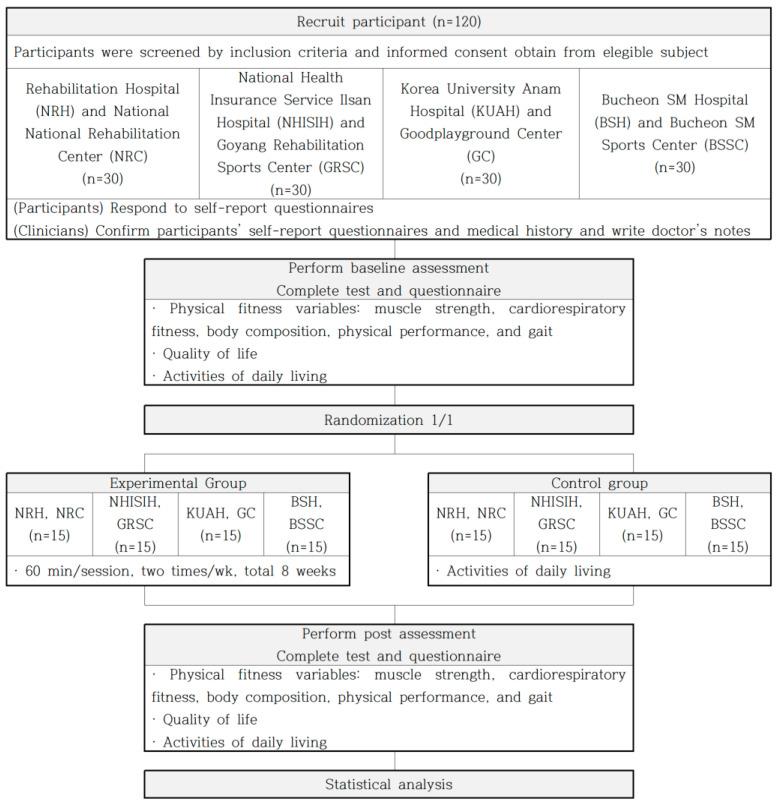
Study flowchart. NRH, National Rehabilitation Hospital; NRC, National Rehabilitation Center; NHISIH, National Health Insurance Service Ilsan Hospital; GRSC, Goyang Rehabilitation Sports Center; KUAH, Korea University Anam Hospital; GC, Good Playground Center; BSH, Bucheon SM Hospital; BSSC, Bucheon SM Sports Center.

This study will be conducted in hospitals (stroke units and outpatient clinics) and rehabilitation centers in the Republic of Korea. A trained researcher who is unaware of the participants’ group assignment will visit the hospitals to obtain informed consent and conduct measurements throughout the study. The National Rehabilitation Center and National Rehabilitation Hospital will initiate and administer the study protocol. This study has been approved by the Medical Ethics Review Committee of the National Rehabilitation Hospital (NRC-2021-05-043), and the protocol follows the SPIRIT 2013 [47] recommendations for clinical trial protocols (identifier: KCT0007521, registered July 19, 2022). Figure 1 shows the study flowchart.

Four teams will conduct this study: (1) the National Rehabilitation Hospital and National Rehabilitation Center; (2) the National Health Insurance Service Ilsan Hospital and Goyang Rehabilitation Sports Center; (3) the Korea University Anam Hospital and Good Playground Center; and (4) the Bucheon SM Hospital and Bucheon SM Sports Center.

### 2.2. Participants

Patients with stroke will be eligible for the trial if they (1) had a stroke (ischemic or hemorrhagic); (2) have been discharged from the hospital after hospitalization for stroke; and (3) voluntarily agreed to participate after understanding the description of the experiment. Patients with stroke will be excluded if they (1) are hospitalized in the hospital; (2) are unable to exercise due to neurological disorders other than stroke (e.g., Parkinson disease), orthopedic problems in the lower extremities, or other cardiopulmonary diseases; (3) are otherwise unable to perform this task based on the judgment of the investigator; and (4) are pregnant.

### 2.3. Randomization of the Participants

After signing the informed consent form, eligible participants referred by each team’s supervisor for the study will be randomized into one of the two trial groups. The sequence will be randomly assigned to the experimental and control groups using a computerized allocation program at a 1:1 ratio. An investigator not involved in participant allocation will prepare this sequence.

### 2.4. Intervention Protocol/Procedure

Participants diagnosed with stroke and discharged from the hospital will be considered for the ReHAPPY study. During the first visit, the staff will notify individuals who qualify for the study. The selection process will be based on whether the patients meet the specific criteria for inclusion and exclusion. Additionally, the staff will clarify the details of the study to the individuals involved, obtain their consent to participate, and provide them with an informed consent form. The participants will then complete a self-report questionnaire and have a face-to-face appointment with the clinician. The clinician will verify each participant’s self-report questionnaire and medical history and issue a doctor’s note. The doctor’s note will be written after reviewing the participant’s responses to the self-report questionnaire and medical history; the note will include the patient’s name, sex, age, diagnosis, date of onset, comorbidities, cautions, and doctor’s opinion.

Before random assignment, two trained instructors will assess the outcome variables of each participant. Afterward, the participants will be randomly assigned to either the control or experimental group. During future evaluations, the participants will be instructed not to disclose their assigned group to the evaluators.

### 2.5. Exercise Trainers and Training

Exercise trainers with at least 5 years of post-stroke rehabilitation experience will conduct the intervention in the experimental group. Furthermore, exercise trainers must undergo a 2 h training session to enhance the consistency of their interventions. They will also be provided with a comprehensive procedure for managing the participants following the trial protocols and recording any adverse events that may occur.

### 2.6. Intervention

Table 1 shows the intervention program, which will be 60 min/session twice weekly for 8 weeks.

The intervention program for the experimental group will consist of 60 min sessions of whole-body exercise, with the duration of each session increasing to a maximum duration of 45 min based on individual ability. The intervention program is a circuit training program that was designed according to the principles of specificity and progressive overload. A program comprising three sets of four to seven movements and exercises that combine aerobic and resistance training has been established. The participants will complete one set of movements. To minimize fatigue, the participants will alternate between upper- and lower-body exercises, and we will allow 1–2 min of recovery time after each set of exercises.

The circuit training combined aerobic and resistance training program will be conducted at 65–80% of the individual’s maximum heart rate measured at baseline. The instructions given to the participants will be to perform the exercise program with an intensity of 12–13 on the rate of perceived exertion scale [48], which is considered “somewhat hard.”

Resistance exercise training will involve the use of a TheraBand (Hygenic Corporation, Akron, OH, USA) for 7 upper-body exercises (shoulder press, seated rows, back row, lat pulldown, chest press, biceps curl, and triceps extension) and 10 lower-body exercises (squat, lunge, deadlift, back extension, bridge, crunch, sit-up, reverse crunch, leg raise, and superman position). The TheraBand’s exercise intensity is indicated by the band’s color. To determine the exercise intensity of the TheraBand, we will use the TheraBand Perceived Exertion Scale for Resistance Exercise with elastic bands [49]. The participants will perform a lateral raise for 15 maximum repetitions (RMs) without separating the upper and lower limbs to select the band color based on the scale [49,50]. The participants will then be asked to perform three sets of 12–15 repetitions for all upper- and lower-body exercises using the TheraBand and power training protocol [51], with a concentric contraction phase implemented as soon as possible, a 1 s pause, and an eccentric contraction phase exceeding 2 s. This power training protocol emphasizes concentric contractions by increasing the speed of motion. It focuses on concentric contractions by increasing movement speed. Aerobic exercise will consist of seven activities (jumping jack, sidestep, pogo jump, knee up, high knee, front step, and back step) that can be performed without equipment or machines.

During the first 2 weeks, the participants will engage in body-weight exercises using their own weight to avoid injury and become accustomed to the training. This approach will be adopted to prevent injury that may arise from intense workouts. During weeks 3–5, participants will use their bands to perform 15 RM exercises. From week 6, the participants will use a higher intensity band. To help the participants with stroke who have difficulty using one hand, a glove will be used to secure the TheraBand during exercise. The intensity of the aerobic exercise will be adjusted by the training instructor based on each participant’s condition. A qualified exercise trainer will supervise the exercise program and monitor the participants’ maximum individual heart rates using a heart rate monitor (Polar, Kempele, Finland) and iPad (Apple, Cupertino, CA, USA). The exercises will gradually be increased in intensity and difficulty with more repetitions and sets. The control group will perform ADLs without any intervention. These participants will be asked to continue with their usual lifestyle habits. They will receive the usual care, including the usual stroke services available to the participants, including but not limited to medical consultations offered by hospitals and rehabilitation services by community-based organizations. The participants in the control group will not receive any specific exercise training from this study scheme.

### 2.7. Outcomes

All the participants will be assessed before the start (baseline) and at the end of the intervention (16 sessions). The outcome measures are listed in Table 2.

#### 2.7.1. Muscle Strength—Isokinetic Muscle Strength

An isokinetic dynamometer (Humac Norm, CSMi, Stoughton, MA, USA) will be used to assess lower extremity strength. An isokinetic dynamometer offers an objective evaluation of concentric dynamic strength [52]. The isokinetic contraction test will be used to assess the peak torque of knee extension and flexion for each lower extremity. During testing, we will ensure that the examined muscle group is isolated and that the extremity and machine axes of rotation are well aligned. The participants will receive special attention in this regard. Before initiating the test, the participants will complete a consistent warm-up routine consisting of two to five submaximal reciprocal concentric extension and flexion movements. Following a resting period of 10 s, the participants will perform five repetitions of knee extension and flexion on both the paretic and non-paretic sides at a speed of 60°/s. After a break of 2–5 min, the participants will perform 15 repetitions of knee extension and flexion on both the paretic and non-paretic sides at a speed of 180°/s. We will record the peak torque of knee extension and flexion on the paretic and non-paretic sides at 60°/s and 180°/s of testing.

#### 2.7.2. Muscle Strength—Grip Strength

A handgrip dynamometer (TKK-5401; Takei Scientific Instruments, Tokyo, Japan) will be used to measure handgrip strength. Stroke professionals commonly assess the strength of a patient’s grip on their paretic and non-paretic hands as a reliable indicator of upper extremity outcomes [53,54]. This measurement method may serve as a substitute for other grip strength assessments. The participants will be instructed to hold the handle tightly for 3 s, and their grip strength will be measured twice (paretic and non-paretic hands), with the mean value recorded.

#### 2.7.3. Cardiorespiratory Fitness—Peak Oxygen Consumption

Cardiorespiratory fitness will be assessed by measuring the peak oxygen consumption [55]. This test is considered the gold standard for patients with stroke because of its objectivity, non-invasiveness, wide usage, and reported effectiveness and safety [53]. All the participants will perform a graded exercise test using a cycle ergometer (Angio CPET with static wall fixation, Lode B.V., Groningen, Netherlands). During the assessment, the participants will wear a facial mask, and their volume of oxygen levels will be monitored using breath gas analysis (K4b2 system, Cosmed, Rome, Italy). The physical fitness levels of the participants will be considered to ensure the safety and effectiveness of the graded exercise test on the cycle ergometer, and the protocol will be set to maintain a consistent 60 rpm [56]. As the participants begin the examination, they will warm up with exercises starting at 0 W for the first 2 min. The workload will then gradually be increased by 10 W every minute. The testing protocol and seat height will be consistent for all the participants before and after the test. The test will continue until the participants decide to stop due to exhaustion. The respiratory exchange ratio will be recorded at the end of the test.

#### 2.7.4. Body Composition—Whole-Body Dual-Energy X-ray Absorptiometry

Body composition will be assessed using dual-energy X-ray absorptiometry with Discovery Wi (Hologic, Waltham, MA, USA). We will record the total and segmental fat-free lean and fat masses.

#### 2.7.5. Body Composition—Bioelectrical Impedance Analysis

Body composition will be assessed using bioelectrical impedance analysis with Inbody S10 (Inbody, Seoul, Republic of Korea). We will collect data on various body composition variables, including skeletal muscle mass, body fat percentage, body mass index, body weight, and waist-to-hip ratio. A stadiometer (BSM370, Inbody) will be used to measure the participants’ height and weight.

#### 2.7.6. Physical Performance—Short Physical Performance Battery

To evaluate lower extremity function, we will use the Short Physical Performance Battery, which consists of three objective physical function tests [57]. These tests measure the time taken to cover 4 m at a comfortable walking speed, the time taken to stand from sitting in a chair five times without halting, and the ability to maintain balance for 10 s in three different foot positions at increasingly difficult levels. For each task, a score between 0 and 4 will be assigned to evaluate the performance, with higher scores denoting improved lower extremity function.

#### 2.7.7. Static Balance—Berg Balance Scale

The Berg Balance Scale (BBS) includes 14 tasks, such as arm stretches, one-legged balancing, and movement. Each task is rated from 0 to 4 points. A score of 56 points is the highest possible score, and the higher the score, the better one’s balance ability [58]. A BBS score of 0–20 points indicates severe balance damage, 21–40 points indicates moderate balance damage, and 41–56 points indicates mild balance damage [58]. The BBS has high relative reliability, with an inter-rater reliability estimated to be 0.97 (95% confidence interval [CI]: 0.96–0.98) and an intra-rater reliability estimated to be 0.98 (95% CI: 0.97–0.99). The absolute reliability of the BBS varies across scales, with minimal detectable changes and 95% CIs varying between 2.8/56 and 6.6/56 [58].

#### 2.7.8. Dynamic Balance—Timed up and Go

During the Timed Up and Go (TUG) test, the participants will stand up from a chair, walk in a straight line for 3 m, turn around, walk back to the chair, and sit down while the examiner records the time to complete the test. The TUG test determines the likelihood of falling. A score below 10 s indicates a minimal risk of falling, whereas a score between 10 and 20 s suggests vulnerability and a heightened risk of falling. A score above 20 indicates a significant risk of falling [59]. The minimum detectable change in stroke is 2.9 s [60].

#### 2.7.9. Gait—10 m Walk Test

The 10 m walk test requires the participant to walk a distance of 10 m twice at a normal walking pace. The time taken (in seconds) will be measured [61], and the mean value will be recorded. The 10 m walk test has a high inter-rater reliability (estimated interclass correlation coefficient [ICC]: 0.87–0.88) and a high inter-rater reliability (ICC: 0.998). The minimum perceptible change is set at 0.05 m/s, and a significant change is set at 0.10 m/s [61].

#### 2.7.10. Secondary Outcomes

The Euro Quality of Life—5 Dimensions questionnaire evaluates personal health status based on five areas: mobility, self-care, typical activities, pain, and anxiety or depression. It consists of multiple-choice questions [62].

The Barthel Index (BI) is a scale that measures performance in ADLs. It consists of 10 variables that describe ADLs. A higher score on the scale indicates greater independence in ADLs. The BI has been proven to have excellent clinometric properties [63].

### 2.8. Participant Timeline

Table 3 shows the enrollment, interventions, and assessment schedule for this study in accordance with the SPIRIT statement [47].

### 2.9. Blinding

Two evaluators who are unaware of the assignment will perform the assessments. The participants cannot be blinded to the assignment due to the nature of the intervention. The instructor conducting the intervention will know the participants’ assignment status and will be instructed not to reveal it at any point during or after the assessment. A data analyst on the research team will input the data into a computer using separate datasheets. This will ensure that the investigators can analyze the data without compromising the privacy of the allocation information.

### 2.10. Data Collection Methods

Before the intervention and during the 16 sessions, the listed outcome measures will be collected, except for the sociodemographic and descriptive variables, which are shown in Table 4.

### 2.11. Data Management and Statistical Analysis

The sample size was determined by referring to a prior study [51]. Before recruiting the participants, we will perform a power analysis using G*Power, version 3.1.9.4 (G*Power software version 3.1.2; University of Kiel, Kiel, Germany) [64]. The overall effect size index for all the outcome measures and the power of the study is 0.5, the probability is 0.05, and the type II error (power of 80%) is minimized. The estimated target sample size is 29 participants on each team; therefore, we will recruit 30 participants on each team, for a total of 120 participants in four teams.

Participant characteristics and comparisons at baseline will be used to evaluate the similarity between the two groups, and a table showing the attributes of the participants assigned to each group will be presented. Discrete variables will be presented as frequencies and percentages. We will use means, standard deviations, medians, and interquartile ranges to summarize continuous variables and time intervals. Both groups will be homogeneous at baseline.

Statistical analyses will be performed using an intention-to-treat approach. Analysis of the primary outcomes will be performed as follows. To determine normality, the dependent variables will be assessed using the Kolmogorov–Smirnov test. The results of the dependent variables will be described using either means and standard deviations or medians and interquartile ranges, depending on the variables’ adjustment to normality. To address the main objective, hypothesis tests will be conducted for every primary outcome, where the alternative hypothesis states that the experimental group is more effective than the control group. Additionally, 95% CIs will be provided for all estimates. For the independent samples in the parametric case, a Student’s t test will be used, whereas for those in the non-parametric case, the Mann–Whitney U test will be used.

Analysis of the secondary outcomes will be performed as follows: for quality of life, contrasts of the hypotheses will be made, and a 95% CI will provide estimates. In addition, it will be helpful to establish a regression model that examines the potential correlations between quality of life and factors such as muscle strength, cardiorespiratory fitness, flexibility, physical performance, static balance, dynamic balance, and gait. To measure the strength of these relationships, we will perform an analysis of variance F-test and individual t-test. The relationship’s strength will be expressed as odds ratios and their 95% CIs.

## 3. Discussion

This randomized, multicenter, parallel-group, double-blind controlled pilot and feasibility trial will be the first to investigate the effectiveness of exercise training programs after patients with stroke are discharged from the hospital with a doctor’s note and referred to a community exercise center in the Republic of Korea. Providing exercise training for stroke care in the community after hospital discharge is supported by best practices [65].

The most common changes caused by stroke are associated with the impairment of the physical fitness components, including muscle weakness, muscle atrophy, decreased muscle power, decreased cardiorespiratory fitness, decreased functional performance, and decreased gait [66]. Physical fitness in these factors is essential, such as in sitting to standing, turning for independent daily living, and stair-climbing activities [67]. Therefore, rehabilitation training to improve physical fitness after stroke onset is necessary to minimize nervous system disability as the nerves (glial cells, neurovascular) recover [26].

A previous study demonstrated that exercise training programs are effective in stroke rehabilitation. Specifically, aerobic and resistance exercises enhance the exercise capacity of patients with stroke [68]. Recent studies have shown that a combination of aerobic and resistance exercises is more beneficial for patients with stroke than aerobic exercise alone [69]. A program consisting of aerobic and resistance exercise, such as the one we will use in this study, is an effective intervention to maintain and improve muscle strength, physical function, cardiorespiratory fitness, and activities of daily living [70,71,72]. Therefore, this exercise training program, which combines resistance and aerobic exercises, will simultaneously improve real-life functions instead of single interventions that only address key functions and need improvement to effectively facilitate these specific functions in patients with stroke.

A systematic review of exercise training programs combining resistance and aerobic exercise in people with stroke found that aerobic exercise was predominantly performed on treadmills and cycle ergometers, whereas resistance exercise was performed using machine-based exercise methods [72]. However, machine-based exercises can lead to injuries [73]. As an alternative, we will use resistance bands for resistance exercise training and body-weight exercises for aerobic exercise training to keep participants safe and injury-free at community exercise centers.

We expect that community-based exercise training will be considered feasible, in that patients with stroke can learn and perform exercise training in any space after being discharged from the hospital and visiting a community exercise center. We also expect the participants to enjoy exercise training and perceive it as beneficial for their recovery. Finally, by conducting an exercise training program based on subject information such as stroke type, degree of disability, medical history, and underlying medical conditions, as well as clinical evaluation results, it is possible to recognize the risk factors that may occur during the exercise training program in advance and contribute to prevention.

This study’s findings could serve as a bridge to facilitate the connection between patients with stroke and community exercise centers after discharge from a hospital in the Republic of Korea. Additionally, the findings of this study will be shared through manuscripts submitted to relevant journals and presented at various stroke-related conferences as well as regional conferences and rounds.

This study will have a limitation in its small sample size for each team (15 participants in the experimental group). It is appropriate to conduct a feasibility study before investing funds and time in a study; however, this may hinder the ability to identify significant differences between the groups.

Considering this feasibility study, our next step is to plan a definitive study to assess the effectiveness of exercise training within the community for improving physical outcomes (muscle strength, cardiorespiratory fitness, physical function, and gait). To pave the way for future studies, the initial outcomes will provide essential insights into crucial factors such as sample size, number of weeks of exercise training, length of exercise training sessions, number per week, control intervention, and primary and secondary outcome measures. Practical information about connecting with community exercise centers after hospital discharge is also essential to inform future proposals. We are currently discussing the establishment of a research collaboration between rehabilitation medicine physicians and exercise specialists to help patients with stroke participate in exercise in the community after hospital discharge. We plan to further expand this area. In the future, we hope to add a home-based exercise training program component (i.e., virtual reality training) to the community.

## 4. Conclusions

This study’s results may add new insights into the effectiveness of community-based exercise training programs after patients with stroke are discharged from the hospital with a doctor’s note and referred to a community exercise center. They may also provide new insights into the ability of patients with stroke to exercise in a community-based setting after hospital discharge. Furthermore, the success of the new exercise training approach could offer valuable information for developing more inclusive protocols for patients with stroke in the future if it proves to be efficacious.

## Figures and Tables

**Table 1:** Intervention protocol.

Warm-Up (10 min)		Main Exercise (40 min)		Cool-Down (5 min)	
Aerobic	Flexibility	Resistance	Aerobic	Cardiovascular	Flexibility
Walking	Static/Dynamic Stretching	Lower Body	Jumping Jack High Knee Sidestep Front step Backstep Knee up Pogo jump	Walking	Static/Dynamic Stretching
		Squat Lunge Bridge			
		Trunk			
		Leg Raise Reverse Crunch Sit-up Crunch Back Extension Superman Position Deadlift			
		Upper Body			
		Chest Press Back Row Shoulder Press Biceps Curl Triceps Extension			

**Table 2:** Outcomes measures.

Outcome Domain	Measures Instrument	T0	T1
Sociodemographic and descriptive data	Ad hoc questionnaire	X	
Primary outcomes			
Muscle strength (Lower and upper extremities)	Isokinetic muscle strength	X	X
	Grip strength	X	X
Cardiorespiratory fitness	VO_2_peak	X	X
	6MWT		
Body composition	Whole-body DEXA	X	X
	BIA	X	X
Physical performance	SPPB	X	X
Static balance	BBS	X	X
Dynamic balance and gait	TUG	X	X
	10 m walk test	X	X
Secondary outcomes			
Quality of life	EQ-5D	X	X
Activities of daily living	Barthel index	X	X
Adverse effects	Open questions		

T0, baseline; T1, evaluation at 16 sessions; 6MWT, 6 min walking test; DEXA, dual-energy X-ray absorptiometry; BIA, bioimpedance analysis; SPPB, Short Physical Performance Battery; BBS, Berg Balance Scale; TUG, Timed Up and Go; EQ-5D, Euro Quality of Life—5 Dimensions.

**Table 3:** Study period: schedule of enrollment, interventions, and assessments of this study.

Study Period				
	Enrollment	Allocation	Post-Allocation	Closeout
Time point, baseline	t	0	T16	tx
Enrollment				
Eligibility screening	X			
Informed consent	X			
(Participant) Self-report questionnaires	X			
(Clinicians) Confirm participants’ self-report questionnaires and medical history and write doctor’s notes	X			
Allocation		X		
Assessments				
Baseline variables		X		
Post-intervention variables			X	X
Intervention				
Experimental group			X	X

**Table 4:** Collection of self-reported questionnaire data, sociodemographic, and descriptive data.

Self-reported questionnaire data	Age	Years
	Sex	Male/female
	Diagnosis	Hemorrhagic/ischemic
	Hypertension	Yes/no
	Anemia	Yes/no
	Dyspnea or Asthma	Yes/no
	Orthostatic hypotension	Yes/no
	Diabetes	Yes/no
	Medications for heart disease	Yes/no
	Coronary stent	Yes/no
	Epilepsy	Yes/no
	Medications for anticoagulant	Yes/no
	Medications for depression	Yes/no
	Acute low back pain within 4 weeks	Yes/no
	Walking due to joint pain	Yes/no
	Medications for osteoporosis	Yes/no
	Spine, hip, or femur fractures due to osteoporosis	Yes/no
	Hip or femur fractures due to falls	Yes/no
Sociodemographic and descriptive data	Blood pressure	mmHg
	Height	cm
	Weight	kg
	Body mass index	Underweight/normal weight/overweight
	Number of cigarettes per day	Number
	Number of drinks per day	Number
	Days of discharge from the hospital	Number of days

## Data Availability

Not applicable.
